# Effect of neuromuscular control exercise program using non-elastic taping on the knee joint in patients with knee osteoarthritis

**DOI:** 10.1097/MD.0000000000042173

**Published:** 2025-04-25

**Authors:** Sang-Woo Yoon, Suhn-Yeop Kim

**Affiliations:** a Department of Physical Therapy, Graduate School, Daejeon University, Daejeon, Republic of Korea; b Department of Physical Therapy, College of Health and Medical Science, Daejeon University, Daejeon, Republic of Korea.

**Keywords:** balance training, elderly, knee osteoarthritis, non-elastic taping, strength training program

## Abstract

**Background:**

This study aimed to investigate the effects of a neuromuscular control exercise program for the knee joint using non-elastic taping on pain, functional disability, quality of life, balance ability, thickness, and contraction ratio of the rectus femoris (RF), vastus medialis (VM), vastus lateralis (VL), and vastus medialis obliquus (VMO) muscles in adults aged 65 years and older with knee osteoarthritis.

**Methods:**

This study was a randomized controlled trial. A total of 44 elderly individuals (aged ≥ 65 years) with knee osteoarthritis were randomly assigned to an experimental group (n = 22) and a control group (n = 22). Both groups underwent a neuromuscular control exercise program, with the experimental group receiving non-elastic taping and the control group receiving sham taping. Repeated measures ANOVA was performed to examine the main effect of time and the interaction effect between time and group.

**Results:**

Both groups showed significant differences in VAS, K-WOMAC, EQ-5D, BBS, muscle thickness, and contraction ratio before and after intervention (*P* < .05). In addition, the experimental group showed more significant differences in the amount of change in K-WOMAC stiffness, K-WOMAC function, EQ-5D, BBS, muscle thickness, and contraction ratio values than the control group.

**Conclusion:**

A neuromuscular control exercise program using non-elastic taping for knee joints is an effective intervention method for improving knee joint pain, function, quality of life, balance ability, muscle thickness and contraction ratio in elderly patients aged ≥ 65 years, with knee osteoarthritis.

## 1. Introduction

Knee osteoarthritis is a highly prevalent chronic condition experienced by approximately 80% of individuals aged 65 years and older.^[1]^ As one of the chronic conditions, knee osteoarthritis leads to changes in joint surface morphology due to aging, as well as damage to surrounding tissues such as ligaments and tendons. This results in inflammation, pain, and decreased joint range of motion.^[2]^ It mainly affects the muscles around the knee, such as the quadriceps femoris, causing muscle imbalance and weakness. Muscles deterioration contributes to the progression of joint instability and symptoms of osteoarthritis.^[3]^ Furthermore, knee osteoarthritis induces pain and joint stiffness, leading to a reduction in the space between the bones and joints, resulting in functional impairment.^[4]^

The knee joint, which bears the body’s weight and enables various movements such as walking and running, experiences accelerated aging.^[5]^ Additionally, the degeneration and deterioration of the knee joint cartilage leads to a decrease in joint mobility.^[6]^ In individuals with knee osteoarthritis, structural changes in the joint worsen pain and impair function, resulting in reduced balance and decreased activity levels.^[7]^ These changes in knee joint structure associated with osteoarthritis contribute to compromised balance, a critical factor in persistent pain and restricted knee joint function, along with decreased muscle strength and joint mobility in patients.^[8]^

Knee osteoarthritis not only causes joint pain and impaired mobility,^[9]^ but also contributes to psychological distress, including anxiety and depression, as patients face increasing restrictions in daily and social activities.^[10]^ The reduced balance ability in knee osteoarthritis patients results in psychological problems such as anxiety and depression related to pain, alongside restrictions on social participation among the elderly. As a result, it negatively impacts the health-related quality of life of patients with knee osteoarthritis patients.^[11]^ Assessment scales for the health-related quality of life of the elderly are essential, as they evaluate physiological, psychological, and socio-cultural factors, as well as their impact on social participation.^[12]^

Knee osteoarthritis weakens the muscles surrounding the knee, leading to muscle imbalance.^[13]^ Consequently, the continuous inhibition of muscles results in changes in muscle composition and structure, leading to a decrease in muscle contraction force, tension, stiffness, and strength. Moreover, incorrect sensory input to the central nervous system causes abnormal movements in the periphery and a loss of proprioceptive sensation.^[14]^ For the evaluation and diagnosis of patients with of knee osteoarthritis patients, accurate and objective assessment criteria for the muscles surrounding the knee are necessary.^[15]^ In clinical practice, ultrasound is primarily used as a noninvasive method for objective evaluation and diagnosis, measuring muscle movement, size, and thickness. Ultrasound examination is a highly reliable tool for muscle measurements.^[16]^

Exercise on an unstable surface can enhance stability and balance based on both fine and gross motor control.^[17]^ Neuromuscular control exercises on unstable surfaces improve functions that are essential for joint stability and proprioceptive feedback. These exercises use more muscles through dynamic adjustments to reduce the joint load and enhance motor coordination.^[18]^ Previous studies have reported the effectiveness of lower limb strength exercises on an unstable surface for improving pain and balance ability in knee osteoarthritis.^[19,20]^ Such improvements in the strength of the muscles around the knee and joint range of motion have a positive impact on pain relief, knee function recovery, and enhancement of balance ability in patients with knee osteoarthritis.^[19]^

A representative intervention method for reducing pain and improving joint range of motion, as well as balance ability, in patients with knee osteoarthritis is the taping technique.^[21]^ The purpose of non-elastic taping technique is primarily to protect against additional damage or stress to injured structures during knee joint rehabilitation.^[22]^ Non-elastic taping applied to the patellar positively impacts pain relief, muscle activation, and increase quadriceps femoris torque.^[23]^ Additionally, it is also known to support the joint and increase biomechanical stability.^[24]^ The non-elastic taping technique has significant effects on stimulating proprioception and neuromuscular control in patients with knee osteoarthritis.^[21]^ As a result, it reduces pain and enhances joint range of motion and strength, promoting improvements in balance ability and proprioception.^[22]^

Research aiming to improve strength and balance in knee osteoarthritis patients to enhance their social participation and quality of life is actively underway.^[25,26]^ However, there is a lack of studies confirming the effectiveness of symptom improvement through non-elastic taping techniques and knee joint neuromuscular control exercise programs in enhancing the strength and balance of elderly individuals with knee osteoarthritis and improving their quality of life. Therefore, the objective of this study was to examine the effects of a knee joint neuromuscular control exercise program using non-elastic taping on pain, functional disability, quality of life, balance ability, and muscle thickness and contraction ratio in elderly patients aged ≥ 65 years with knee osteoarthritis. The goal was to provide evidence for the utility of non-elastic taping and its valid clinical application. This study is important in that it provides a comparative evaluation of the application of non-elastic taping techniques combined with neuromuscular control exercise program. By analyzing the effects of this approach on knee osteoarthritis, it can provide evidence of the intervention’s effectiveness and its clinical applicability.

The hypotheses of this study were’ as follows: First, the non-elastic taping group (experimental group) will show significant changes in pain levels, functional levels, quality of life, balance ability, and contraction ratio before and after the intervention. Second, the sham taping group (control group) will also exhibit changes in these variables before and after the intervention. Third, there will be significant differences in these changes between the experimental and control groups.

## 2. Methods

### 2.1. Participants

This study examined 52 participants from Y Hospital in S City, who voluntarily agreed to participate as out-patients with knee pain after receiving a detailed explanation of the procedures and purpose of the study. The intervention period was from November 2023 to April 2024. Inclusion criteria included individuals who were 65 years of age or older, had a visual analog scale (VAS) score of 3 or higher, and had been diagnosed with knee osteoarthritis by a specialist. Exclusion criteria included individuals who had experienced lower limb trauma within the past 3 weeks, had neurological symptoms and impairments in the lower limbs, had medical issues related to the cardiovascular system, or had been diagnosed with Kellgren-Lawrence Grade (K-L Grade) 3 or higher for knee osteoarthritis by a specialist. This study was approved by the Ethical Committee of Daejeon University (no. 1040647-202312-HR-005-03) and registered in the World Health Organization (WHO) International Clinical Trials Registry Platform (no. KCT0009280).

### 2.2. Study design

The study procedures and purposes were explained to the participants. Only those who provided written consent to participate were included in this study. This was a randomized controlled trial conducted. The sample size was determined using the G*Power program (G-Power, University of Kiel, Kiel, Germany). Assuming a main effect size (*d*) of 0.82 based on Kim et al,^[19]^ an alpha level (α) of 0.05, and a power (1 − β) of 0.80, a total of 20 participants per group were required, with a dropout rate of 10%, resulting in a minimum of 22 participants per group.^[27]^ Among the 52 recruited participants, 8 participants including those with a K-L grade of 3 or higher (n = 4) and those who scored below 3 on the pain level (VAS) (n = 4), were excluded from the study. After conducting pretests on the remaining 44 participants, they were randomly assigned to either the experimental or the control group. Random assignment was performed using an online randomization program (http://www.randomizer.org) to allocate participants to the experimental group (n = 22) or control group (n = 22), under the supervision of the researcher.

**Figure 1.**
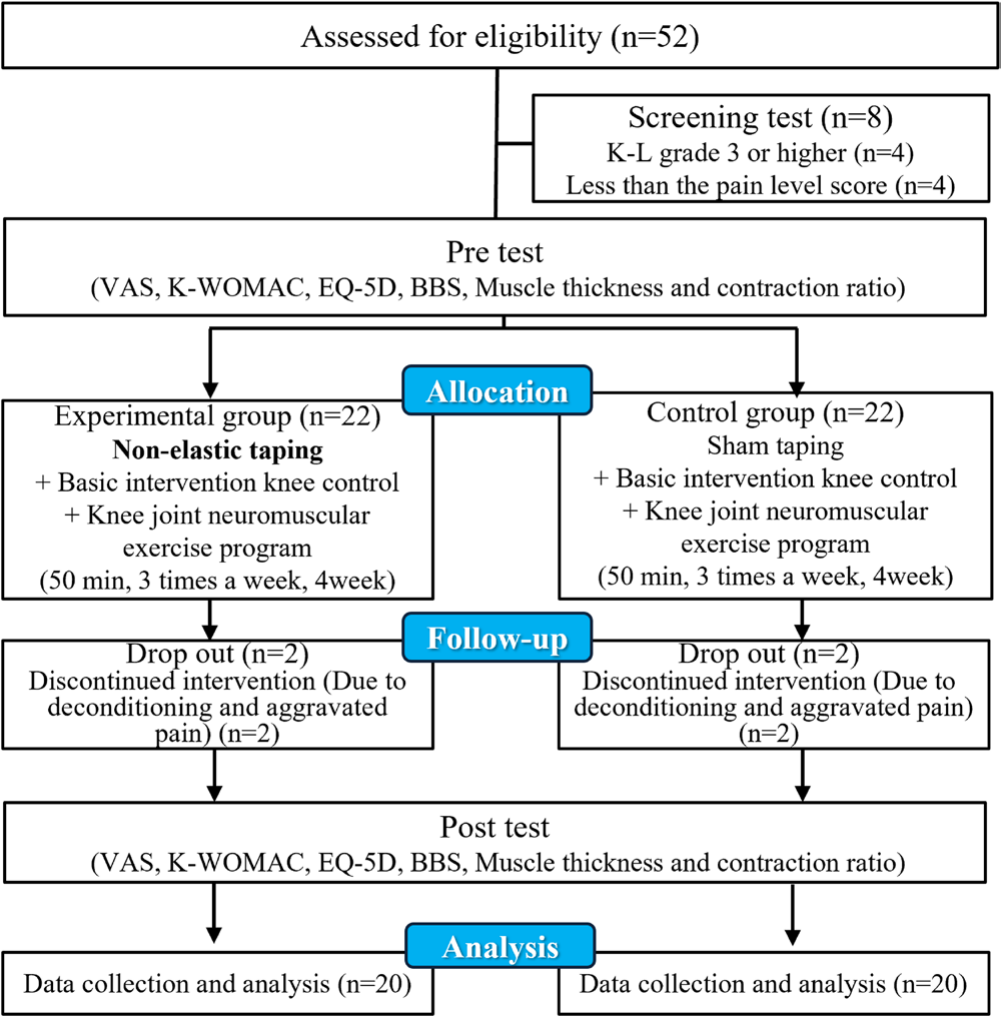
CONSORT flow chart.

Both groups participating in the study performed a knee joint neuromuscular control exercise program. Additionally, in the experimental group, non-elastic taping was applied, whereas the control group received sham taping. To assess the effects of the knee joint neuromuscular control exercise program with taping techniques, measurements were taken before and after the intervention using the VAS, the Korean version of the Western Ontario and McMaster universities arthritis index (K-WOMAC), the euro quality of life 5 dimension (EQ-5D), the berg balance scale (BBS), and the muscle thickness and contraction ratio. The design of this study is illustrated as follows (Fig. 1).

### 2.3. Interventions

Both groups received basic physical therapy consisting of hot pack (Sambu Medical, Daejeon, Korea) and interferential current therapy (ProMed III, StraTek, Korea) applied to the knee area. Sambu Illite helps to alleviate muscle stiffness, promotes blood circulation, increases tissue flexibility, and reduces pain. Interferential current therapy is effective in reducing pain. Both Sambu Illite and interferential current therapy were applied for 10 minutes per session before exercise, 3 times a week for a total of 4 weeks.

**Figure 2.**
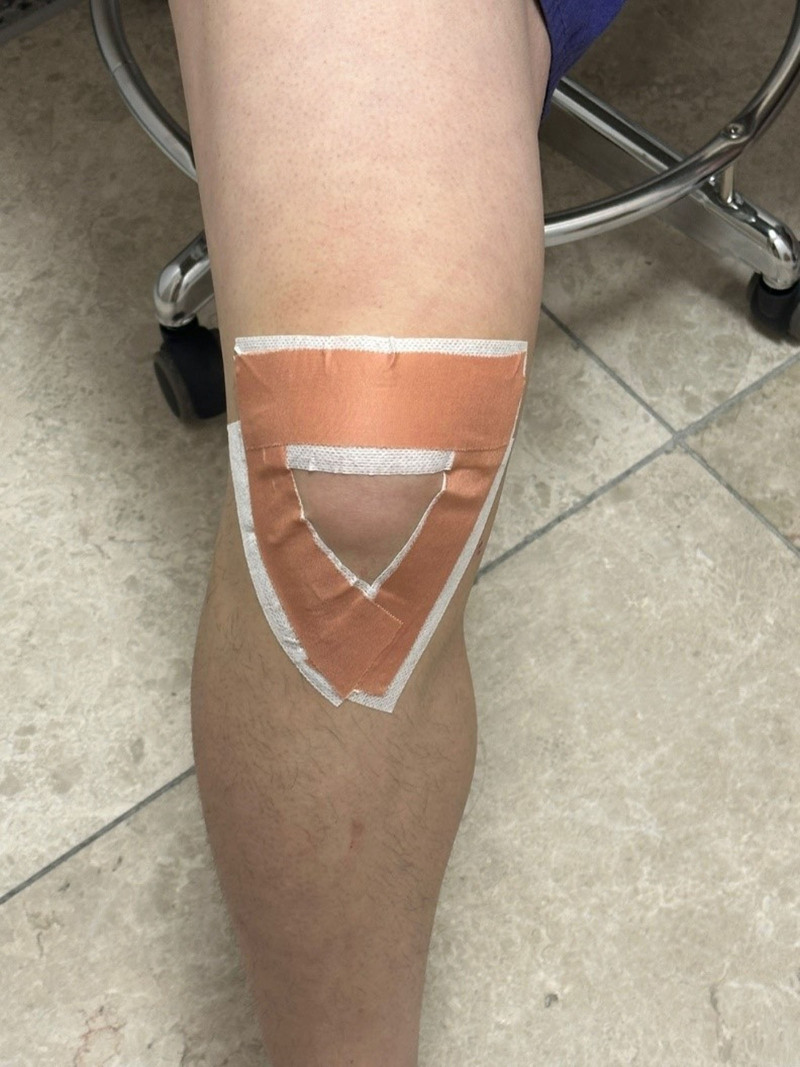
Non-elastic taping to pull skin and soft tissue into the center of the knee.

Non-elastic taping applied to the experimental group used Endura tape (3.83 cm; Endura, Shanghai, China), while protective taping used Endura Fix tape (5 cm; Endura, Sydney, NSW, Australia). Non-elastic taping was applied with the participant’s knee joint bent at a 30-degree angle (Fig. 2).

1) The application the V-shaped taping started from the rough surface of the tibia and was attached to the gaps of the inner and outer knee joints. The taping was applied with the therapist’s hands gathering the skin and connective tissue towards the center of the knee.
2) The upper taping started from the lateral side of the knee and was taped horizontally, applying pressure to the upper part of the knee to induce a backward tilt of the knee cap, while taping.

**Figure 3.**
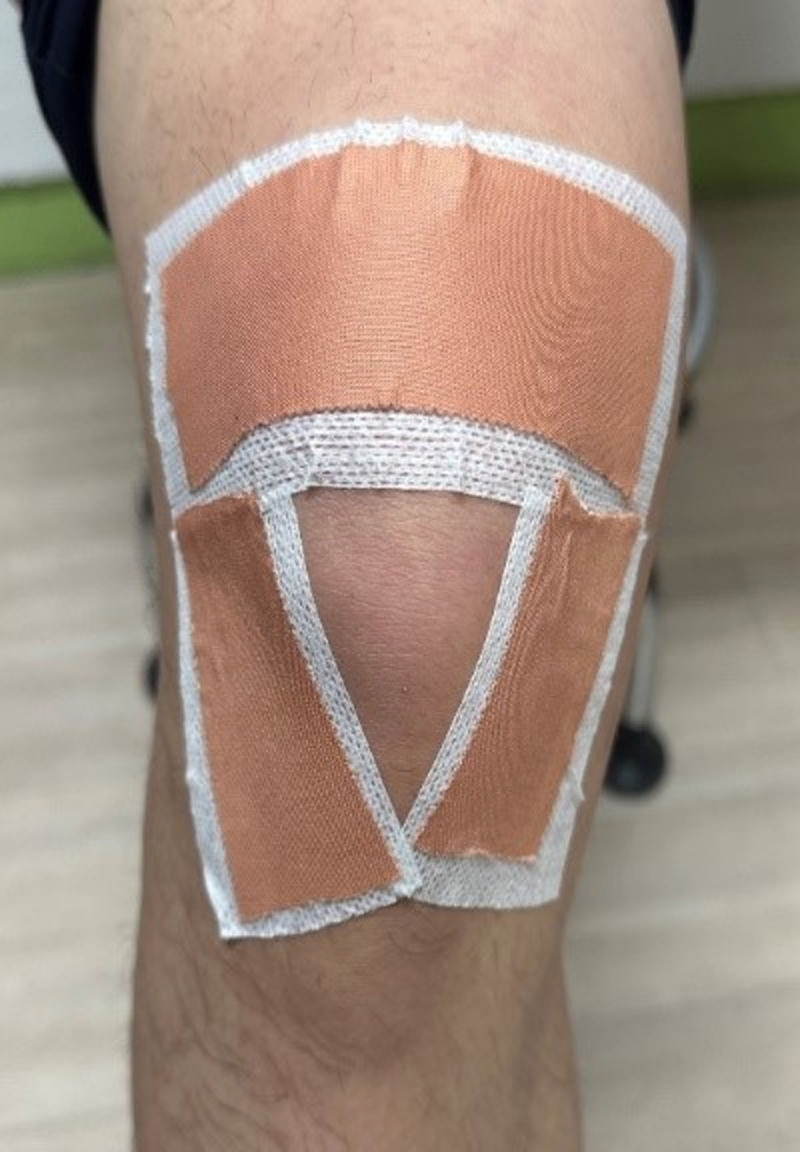
Sham taping to compare with non-elastic taping.

Sham taping was applied to the control group by using the same tape on the same area. The tape was attached without overlapping the ends to minimize its impact on the knee joint. Additionally, it was applied without causing movement or exerting pressure on the skin, soft tissue, or the knee bone (Fig. 3).

Both groups underwent a knee joint neuromuscular control exercise program, which was adapted and enhanced based on the knee joint neuromuscular control exercise program conducted in the study by Kim et al.^[19]^ The exercise program was performed barefoot on an aero-step (Aero-Step^®^ XL; TOGU, Bavaria, Germany). The exercises aimed to induce stability around the knee by strengthening the quadriceps femoris and lower limb muscles and consisted of one-leg stand, marching in place, squat, weight shift, and weight shifting in squat stance. Preparatory and cool-down stretches were provided for 5 minutes each before and after the intervention, and each training program was performed for 3 sets of 20 seconds with a 30-second rest interval between sets. The intervention was applied for approximately 30 minutes per session, 3 times a week, for a total of 4 weeks.

### 2.4. Assessment methods and instruments

Participants’ pain was assessed using the VAS. The VAS consists of a 10 cm line. Pain intensity was rated on a scale from 0 (no pain at all) to 10 (worst pain imaginable), with participants marking their level of pain experienced during a 10-m walk. The measurement method involved participants indicating their current pain level on the line, and the score was recorded accordingly. The reliability of this test was reported as *r* = 0.97 in the study by.^[28]^

Participants’ functional status of the knee joint was assessed using the K-WOMAC index. This evaluation tool is a self-administered questionnaire designed to assess pain and functional status in patients with hip or knee osteoarthritis. It consists of 24 items, divided into 3 domains: pain (5 items), stiffness (2 items), and function (17 items). Pain items evaluate the patient’s state during rest or physical activity.^[29]^ Each question was rated on a Likert scale, with (0 = none, 1 = mild, 2 = moderate, 3 = severe, and 4 = very severe). The score range for each domain was as follows: pain items ranged from 0 to 20 points, stiffness items from 0 to 8 points, and physical function items from 0 to 68 points, totaling 96 points. The intraclass correlation coefficient for the reliability of this assessment tool ranges from 0.79 to 0.89, and the internal consistency Cronbach’s alpha was 0.97, indicating a high level of reliability.^[30]^

To assess the participants’ quality of life, the EQ-5D was used in this study. This assessment tool is widely employed in the healthcare field to measure and evaluate health-related quality of life.^[31]^ The EQ-5D is divided into 5 domains: anxiety/depression, self-care, pain/discomfort, usual activities, and mobility. Each domain consists of items structured as multiple-choice questions. Participants rated their current health status for each domain on a 3-level scale using this questionnaire, and this assessment tool demonstrated high reliability, with a *r* value of 0.87.^[32]^

The BBS was used to assess the participants’ balance ability. The BBS is a scale that evaluates both static and dynamic balance abilities, divided into 3 areas: sitting, standing, and changes in posture, with a total of 14 detailed items.^[33]^ It is scored on a 5-point scale ranging from 0 to 4, with a total score of 56. The intra-rater reliability of the BBS was reported to be *r* = 0.99, and the inter-rater reliability was *r* = 0.98, indicating a high reliability.^[34]^

To measure the thicknesses of the rectus femoris (RF), vastus medialis (VM), vastus lateralis (VL), and vastus medialis obliquus (VMO) around the knee joint, measurements were conducted using the M-mode setting of an ultrasound device (MySonoU6, Samsung, Seoul, Korea). Following the criteria of a study classified by the dominant direction when ascending stairs,^[35]^ all participants’ dominant leg was identified as the right leg. Consequently, the participants were positioned lying on a measurement table with their right leg fully extended for measurement. Quadriceps muscle thickness was measured in the following order: RF, VM, VL, and VMO. The RF measurement position was 50% along an imaginary line connecting the superior pole of the patella to the anterior superior iliac spine (ASIS). For VM, measurements were taken 20% above the superior pole of the patella and 12.5% medially along the circumference of the muscle. VL measurements were performed 10% laterally along the circumference of the RF. The VMO thickness was measured 4 cm above and 3 cm medial to the superior pole of the patella. Muscle thickness was measured during both relaxation and contraction, and the contraction ratio was calculated as the ratio of muscle thickness during contraction to that during relaxation. The inter-rater reliability of muscle thickness measurement using ultrasound has been reported to be very high (*r* = 0.99).^[36]^

### 2.5. Data analysis

Data analysis was conducted using SPSS software (version 25.0; IBM, Chicago). Descriptive statistics were used to analyze the general characteristics of the participants, and the measured variables were presented as means and standard deviations. The Shapiro–Wilk test was used to assess normality, and the chi-square test was conducted for gender analysis. Repeated measures ANOVA were performed to examine the main effect of time and the interaction effect between time and group. Additionally, Cohen’s *d* was calculated to determine the effect size. The level of significance was set at *P* < .05.

## 3. Results

Data of 20 participants in the experimental group and 20 participants in the control group among a total of 44 subjects were collected. Four participants dropped out of the study due to deconditioning and pain aggravation. The general characteristics of the participants are presented (Table 1).

**Table 1:** General characteristics.

Variables	Experimental group (n = 20)	Control group (n = 20)	*t*/χ^2^	*P*
Sex (male/female)	4/16*	3/17	−0.406	.687
Age (year)	71.15 ± 3.59†	71.70 ± 3.81	−0.472	.640
Height (cm)	159.46 ± 6.16	159.57 ± 6.64	−0.069	.945
Weight (kg)	63.06 ± 7.48	63.96 ± 6.60	−0.403	.689
BMI (kg/m²)	24.80 ± 2.42	25.13 ± 2.38	−0.445	.659

BMI = body mass index.

* Count.

† Mean ± standard deviation.

Both the experimental and control groups were homogeneous for VAS, K-WOMAC, EQ-5D, and BBS scores in the pretest. Both groups exhibited significant differences before and after the intervention (*P* < .05). Variations in K-WOMAC stiffness, K-WOMAC function, EQ-5D, and BBS scores differed significantly between the groups (*P* < .05). All dependent variables showed change depending on the measurement time (*P* < .05) and K-WOMAC stiffness, K-WOMAC function, and BBS there were interaction of time × group for measurement times (*P* < .05) (Table 2).

**Table 2:** The results of two-way ANOVA of pain, functional level, quality of life and balance ability before and after the intervention between groups.

Variables		Experimental group (n = 20)	Control group (n = 20)	time F (*P*)	time*group F (*P*)	Cohen’s d (Effect size)
VAS (score)	Baseline	6.90 ± 1.16*	6.65 ± 1.42			
	Post	4.15 ± 1.18	4.25 ± 1.16	88.623 (.000)	0.217 (.644)	2.35
	Change	−2.65 ± 1.50	−2.40 ± 1.87			
K-WOMAC pain (score)	Baseline	16.25 ± 3.53	15.90 ± 3.67			
	Post	13.85 ± 3.28	13.65 ± 3.20	108.113 (.001)	0.013 (.909)	0.70
	Change	−2.40 ± 2.41	−2.25 ± 5.32			
K-WOMAC stiffness (score)	Baseline	6.45 ± 1.70	6.30 ± 1.69			
	Post	4.45 ± 1.36	6.05 ± 1.32	8.301 (.006)	5.022 (.031)	1.29
	Change	−2.00 ± 2.34	−.25 ± 2.59			
K-WOMAC function (score)	Baseline	61.60 ± 10.15	59.55 ± 9.96			
	Post	47.55 ± 8.61	54.55 ± 9.42	130.720 (.000)	29.502 (.000)	1.49
	Change	−14.05 ± 7.01	−5.00 ± 2.51			
K-WOMAC total (score)	Baseline	84.30 ± 12.86	81.75 ± 10.07			
	Post	65.85 ± 8.79	74.25 ± 9.66	116.742 (.000)	20.786 (.000)	1.67
	Change	−18.45 ± 8.25	−7.50 ± 6.88			
EQ-5D (score)	Baseline	0.67 ± .13	0.69 ± .15			
	Post	0.79 ± .08	0.80 ± .09	66.744 (.000)	0.127 (.723)	−1.11
	Change	0.12 ± .07	0.10 ± .09			
BBS (score)	Baseline	33.35 ± 5.73	34.90 ± 6.03			
	Post	45.05 ± 5.20	40.75 ± 5.10	135.976 (.000)	15.108 (.000	−2.13
	Change	11.70 ± 5.75	5.85 ± 3.49			

BBS = berg balance scale, EQ-5D = euro quality of life 5 dimension, K-WOMAC = Korean version of the Western Ontario and McMaster universities arthritis index, VAS = visual analogue scale.

* Mean ± Standard deviation.

Both the experimental and control groups were homogeneous in terms of muscle thickness and contraction ratio in the pretest. Both groups exhibited significant differences before and after the intervention (*P* < .05). Variations in muscle thickness and contraction ratio differed significantly between the groups (*P* < .05). The dependent variables of muscle thickness and contraction ratio showed changes depending on the measurement time (*P* < .05). Furthermore, muscle thickness and contraction ratio there were interaction of time × group for measurement times (*P* < .05) (Table 3).

**Table 3:** The results of two-way ANOVA of muscle thickness and contraction ratio before and after the intervention between groups.

Muscle thickness		Experimental group (n = 20)	Control group (n = 20)	time F (*P*)	time*group F (*P*)	Cohen’s *d* (Effect size)
RF in resting (mm)	Baseline	31.07 ± 3.73*	31.08 ± 3.21			
	Post	31.15 ± 3.69	31.18 ± 3.21	2.539 (.119)	0.050 (.825)	−0.02
	Change	0.08 ± 0.25	0.10 ± 0.45			
VL in resting (mm)	Baseline	34.02 ± 3.57	34.01 ± 3.13			
	Post	33.99 ± 3.57	34.01 ± 3.12	2.182 (.148)	4.057 (.051)	0.01
	Change	−0.04 ± 0.010	−0.01 ± 0.02			
VM in resting (mm)	Baseline	33.01 ± 3.54	32.98 ± 3.02			
	Post	33.05 ± 3.55	32.99 ± 3.02	1.551 (.221)	0.332 (.568)	−0.01
	Change	0.04 ± 0.19	0.01 ± 0.04			
VMO in resting (mm)	Baseline	15.06 ± 1.73	15.08 ± 1.52			
	Post	15.06 ± 1.71	15.09 ± 1.52	0.220 (.642)	0.948 (.336)	0
	Change	−0.01 ± 0.05	0.01 ± 0.04			
RF in contraction (mm)	Baseline	31.42 ± 3.62	31.43 ± 3.18			
	Post	33.55 ± 3.42	32.05 ± 3.27	21.632 (.000)	6.821 (.013)	−0.60
	Change	2.12 ± 2.53	0.62 ± 0.50			
VL in contraction (mm)	Baseline	34.77 ± 3.55	34.73 ± 3.14			
	Post	37.45 ± 3.50	35.31 ± 3.20	358.810 (.000)	150.102 (.000)	−0.76
	Change	2.69 ± 0.73	0.59 ± 0.23			
VM in contraction (mm)	Baseline	33.67 ± 3.55	33.61 ± 3.01			
	Post	36.75 ± 3.41	34.70 ± 3.02	4356.494 (.000)	997.216 (.000)	−0.88
	Change	3.09 ± 0.26	1.09 ± 0.10			
VMO in contraction (mm)	Baseline	15.56 ± 1.74	15.62 ± 1.53			
	Post	18.11 ± 1.72	16.99 ± 1.41	3789.091 (.000)	348.494 (.000)	−1.47
	Change	2.55 ± 0.08	1.36 ± 0.27			
RF contraction ratio	Baseline	1.02 ± 0.01	1.01 ± 0.01			
	Post	1.08 ± 0.08	1.03 ± 0.01	24.885 (.000)	8.958 (.005)	−1.05
	Change	0.09 ± 0.07	0.00 ± 0.00			
VL contraction ratio	Baseline	1.02 ± 0.00	1.02 ± 0.00			
	Post	1.10 ± 0.03	1.04 ± 0.01	273.249 (.000)	114.195 (.000)	−3.77
	Change	0.10 ± 0.02	0.00 ± 0.00			
VM contraction ratio	Baseline	1.02 ± 0.00	1.02 ± 0.00			
	Post	1.11 ± 0.02	1.05 ± 0.01	730.335 (.000)	164.160 (.000)	−6.36
	Change	0.10 ± 0.00	0.01 ± 0.02			
VMO contraction ratio	Baseline	1.03 ± 0.02	1.04 ± 0.01			
	Post	1.20 ± 0.03	1.13 ± 0.03	1401.048 (.000)	117.199 (.000)	−6.66
	Change	0.18 ± 0.04	0.10 ± 0.02			

RF = rectus femoris, VL =vastus lateralis, VM = vastus medialis, VMO = vastus medialis obliquus.

* Mean ± Standard deviation.

## 4. Discussion

This study was conducted to investigate the effects of adding non-elastic taping to a knee joint neuromuscular control exercise program in patients with knee osteoarthritis. The experimental group, which underwent at knee joint neuromuscular control exercise program with non-elastic taping, showed significant differences in K-WOMAC stiffness, K-WOMAC function, EQ-5D, BBS, muscle thickness during contraction, and contraction ratio.

Knee osteoarthritis is a chronic condition that occurs in the knee joints, which bear a significant mechanical burden on the body, leading to stiffness and functional impairment of the joints. Lee and Heo^[37]^ reported a significant decrease in K-WOMAC function (effect size, *d* = 1.27) and K-WOMAC stiffness (effect size, *d* = 0.62) after applying quadriceps strengthening therapy with the taping method for 4 weeks in individuals aged 60 years and older with knee osteoarthritis (*P* < .05). Additionally, Hwang et al^[38]^ observed a significant decrease in K-WOMAC total (effect size, *d *= 1.14) after applying a 12-week knee joint rehabilitation exercise program in individuals aged 70 years and older with knee osteoarthritis (*P* < .05). Consistent with previous studies, our study found a significant decrease in all K-WOMAC items before and after the intervention in all participants (*P* < .05). Additionally, both groups showed decreases, but there was also a significant difference between the experimental group that underwent non-elastic taping and the control group that underwent sham taping in terms of K-WOMAC function scores, which decreased from 61.60 to 47.55 points, representing an average reduction of 14.05 points (effect size, *d* = 1.49), and K-WOMAC stiffness scores, which decreased from 6.45 to 4.45 points, representing an average reduction of 2.00 points (effect size, *d* = 1.32). compared to the K-WOMAC function scores from 59.55 to 54.55 points, averaging 5.00 points (effect size, *d* = 0.52), and K-WOMAC stiffness scores from 6.30 to 6.05 points, averaging 0.25 points (effect size, *d* = 1.06) (*P* < .05). This suggests that the non-elastic taping applied by the experimental group reduced the external forces and loads on the knee joint, leading to a positive impact on the knee joint. As a result, it is believed that stiffness in the knee joint decreases, leading to functional improvement in the knee joint.

As aging progresses, the most critical symptom of physical decline is impaired balance ability.^[39]^ Balance impairment has a detrimental effect on older adults with knee osteoarthritis throughout their daily lives. Park and Kim^[8]^ reported a significant improvement in balance ability after 4 weeks of lower limb strength and balance exercises in adults aged 65 years and older with chronic knee osteoarthritis (*P* < .05, effect size, *d* = 0.63). Additionally, Kim and Choi^[40]^ found a significant improvement in balance after stabilization exercises using a Swiss ball and whole-body vibration in women aged 70 years and older with knee osteoarthritis (*P* < .05, effect size, *d* = 2.01). In this study, all participants showed an improvement in balance ability after the intervention (*P* < .05). Additionally, both groups showed increases, but there was also a significant difference between the experimental group (effect size, *d* = 2.09) with non-elastic taping and the control group (effect size, *d* = .98) (*P* < .05). This suggests that non-elastic taping applied to the experimental group had a positive effect on balance improvement, possibly by enhancing the strength of the muscles around the knee joint and stimulating proprioceptive sensory receptors, leading to improved sensory feedback mechanisms and shortened muscle response time, ultimately contributing to increased balance ability.

Elderly individuals with knee osteoarthritis experience a decreased quality of life due to limitations in daily activities caused by muscle imbalance around the knee joint, muscle weakness, reduced range of motion, and increased pain. Bennell et al^[41]^ reported an improvement in quality of life in individuals aged 60 years and older with knee osteoarthritis after providing a 25-week progressive home exercise program (*P* < .05, effect size, *d* = 1.00). Additionally, Park and Lee^[28]^ reported an improvement in quality of life in individuals aged 65 years and older with knee osteoarthritis after applying kinesio taping and joint mobilization for 4 weeks (*P* < .05, effect size, *d* = 0.83). In this study, all participants showed improvement in quality of life indicators before and after the intervention, yielding results similar to those of previous studies (*P* < .05). Additionally, both groups showed increases, but there was also a significant difference between the experimental group applying non-elastic taping (effect size, *d* = 1.01) and the control group (effect size, *d* = .80) (*P* < .05). This suggests that non-elastic taping has a beneficial effect on knee joint function and pain reduction, leading to improved participation in daily activities and social engagement, thereby enhancing quality of life indicators.

Elderly individuals with knee osteoarthritis experience a gradual decline in the strength and mass of muscles around the knee, leading to a decrease in balance ability and physical activity capacity. Choi et al^[42]^ reported a significant improvement in quadriceps thickness after applying knee joint flexion exercises for 8 weeks in individuals aged 70 years and older with knee osteoarthritis (*P* < .05). Additionally, Park and Park^[43]^ demonstrated an increase in quadriceps thickness around the knee joint after whole-body vibration exercises with weighted vests for 8 weeks in elderly individuals aged 65 years and older (*P* < .05). In this study, significant improvements were observed in all parameters, except muscle thickness during relaxation (*P* < .05). Additionally, both groups showed increases, but there was also a significant difference between the experimental group that underwent non-elastic taping and the control group that underwent sham taping in terms of RF contraction (effect size, *d* = 0.55), VM contraction (effect size, *d* = 0.88), VL contraction (effect size, *d* = 0.76), and VMO contraction (effect size, *d* = 1.47) compared to the control group that underwent sham taping, where the effect sizes were RF (*d* = 0.19), VM (*d* = 0.36), VL (*d* = 0.18), and VMO (*d* = .93) (*P* < .05). This suggests that non-elastic taping enhances knee joint stability and proper alignment through functional support, thereby positively influencing neuromuscular control.

This study aimed to investigate the effects of a non-elastic taping-based knee joint neuromuscular control exercise program in patients aged 65 years and older with knee osteoarthritis. However, this study had several limitations. First, the majority of female patients, with an average age of approximately 71 years, were more prevalent than male patients, making it difficult to generalize the effects of exercise. Second, the intervention period was limited to 4 weeks, making it challenging to assess additional effects after 4 weeks. Third, knee joint neuromuscular control exercises appear to have short-term effects, and longer-term exercise of over 6 months may be necessary for sustained effects in patients with knee osteoarthritis. Future research should address and overcome these limitations to continue investigating intervention methods that can improve the quality of life of elderly patients.

## 5. Conclusions

This study was conducted to investigate the effects of a non-elastic taping-based knee joint neuromuscular control exercise program on knee joint pain, functional disability, quality of life, balance ability, muscle thickness, and contraction ratio in patients aged 65 years and older with knee osteoarthritis. The results showed that the experimental group, which underwent a knee joint neuromuscular control exercise program using non-elastic taping, demonstrated significant improvements in knee joint functional disability levels, quality of life, balance ability, and muscle thickness and contraction ratio during contraction. Based on these findings, it can be concluded that a knee joint neuromuscular control exercise program using non-elastic taping is an effective intervention method for patients aged 65 years and older with knee osteoarthritis.

## Acknowledgments

The authors would like to express their sincerest gratitude to the patients for their participation, thereby enabling the acquisition of data.

## Author contributions

**Conceptualization:** Sang-woo Yoon.

**Data curation:** Sang-woo Yoon.

**Formal analysis:** Sang-woo Yoon.

**Methodology:** Sang-woo Yoon, Suhn-Yeop Kim.

**Supervision:** Suhn-Yeop Kim.

**Visualization:** Sang-woo Yoon, Suhn-Yeop Kim.

**Writing – original draft:** Sang-woo Yoon.

**Writing – review & editing:** Suhn-Yeop Kim.
